# A Novel Three-Colour Fluorescence *in Situ* Hybridization Approach for the Detection of t(7;12)(q36;p13) in Acute Myeloid Leukaemia Reveals New Cryptic Three Way Translocation t(7;12;16)

**DOI:** 10.3390/cancers5010281

**Published:** 2013-03-11

**Authors:** Abdulbasit Naiel, Michael Vetter, Olga Plekhanova, Elena Fleischman, Olga Sokova, Grigory Tsaur, Jochen Harbott, Sabrina Tosi

**Affiliations:** 1 Leukaemia and Chromosome Research Laboratory, Division of Biosciences, Brunel University, London, Middlesex UB8 3PH, UK; E-Mail: abdulbasit.naiel@brunel.ac.uk; 2 MetaSystems, Altlussheim 68804, Germany; E-Mail: mvetter@metasystems.de; 3 Regional Children’s Hospital N 1, Ekaterinburg 620149, Russia; E-Mails: oliagrom@mail.ru (O.P.); tsaur@mail.ru (G.T.); 4 N.N. Blokhin Russian Cancer Research Center Russian Academy of Medical Science, Moscow 115478, Russia; E-Mails: elefle@gmail.com (E.F.); flesok@yandex.ru (O.S.); 5 Research Institute of Medical Cell Technologies, Ekaterinburg 620149, Russia; 6 Oncogenetic Laboratory, Department of Paediatric Haematology and Oncology, Justus Liebig University, Giessen 35392, Germany; E-Mail: jochen.harbott@paediat.med.uni-giessen.de

**Keywords:** fluorescence *in situ* hybridization, chromosomal translocations, childhood leukaemia, t(7;12), *ETV6*, *HLXB9*

## Abstract

The t(7;12)(q36;p13) translocation is a recurrent chromosome abnormality that involves the *ETV6* gene on chromosome 12 and has been identified in 20–30% of infant patients with acute myeloid leukaemia (AML). The detection of t(7;12) rearrangements relies on the use of fluorescence *in situ* hybridization (FISH) because this translocation is hardly visible by chromosome banding methods. Furthermore, a fusion transcript *HLXB9-ETV6* is found in approximately 50% of t(7;12) cases, making the reverse transcription PCR approach not an ideal screening method. Considering the report of few cases of variant translocations harbouring a cryptic t(7;12) rearrangement, we believe that the actual incidence of this abnormality is higher than reported to date. The clinical outcome of t(7;12) patients is believed to be poor, therefore an early and accurate diagnosis is important in the clinical management and treatment. In this study, we have designed and tested a novel three-colour FISH approach that enabled us not only to confirm the presence of the t(7;12) in a number of patients studied previously, but also to identify a cryptic t(7;12) as part of a complex rearrangement. This new approach has proven to be an efficient and reliable method to be used in the diagnostic setting.

## 1. Introduction

Chromosomal abnormalities are a hallmark of cancer, with numerous examples of non-random translocations found in leukaemia [1,2]. Recurrent chromosomal abnormalities are used in the clinical practice for their diagnostic and prognostic value, their association with specific subtypes and subsequently as parameters on which to base therapy decisions. A number of genes with a pivotal role in leukaemia have been found rearranged with several partners in different cases [3,4,5]. One of these genes is the ets variant 6 gene, *ETV6*, that has been reported to be rearranged with approximately 48 chromosomal bands in different types of abnormality. Several mechanisms of actions have been proposed for this gene in leukaemogenesis, showing an array of behavioural patterns depending on the partner involved [6]. 

The t(7;12)(q36;p13) has been identified as a recurrent abnormality in a subset of paediatric AML. However, there are few reports of t(7;12) found in childhood ALL (see Table 1). This rearrangement involves the *ETV6* gene in 12p13 and variable breakpoints on 7q36 [7,8,9,10], usually proximal to the homeobox HB9 gene, *HLXB9*.

Several reports have investigated the incidence of the t(7;12) in infant leukaemia and have shown that this rearrangement occurs in approximately one third of paediatric patients with age between 0–2 years [9,10,11,12]. According to these studies, the t(7;12) has not been associated with any particular AML subtype and constitutes a poor prognostic factor. From the cytogenetic point of view, the t(7;12) has been found as a sole abnormality in 2 out of 44 cases reported to date (see Table 1 for a summary of the literature). In the majority of cases, this rearrangement was accompanied by the presence of other abnormalities, mainly numerical. The presence of an extra chromosome 19 was found in the majority of cases, with an incidence of 33 out of 44 patients. This additional chromosome was also accompanied by the presence of an extra chromosome 8 (in 10 of these cases), in the same (8 cases) or different clones (2 cases), or an extra chromosome 13 (2 cases). The presence of these additional abnormalities in such a consistent fashion has prompted the screening for t(7;12) cases on infant patients selected on the basis of having a +19 and/or +8 in their leukaemic cells [9,10,12].

The mechanism of oncogenesis at the basis of t(7;12) leukaemias remains to be elucidated. Reverse-Transcription PCR experiments have shown that this translocation results in a fusion transcript between exon 1 of the *HLXB9* gene, localized in 7q36, and exon 3 of *ETV6* [13]. However, studies on larger series of patients have shown that such a fusion transcript is present only in approximately 50% of t(7;12) leukaemias [12,14]. To date, there is no report that confirms the presence of a chimeric protein in the positive cases. Nevertheless, all of the t(7;12) patients have shown an ectopic expression of *HLXB9*, both at transcript and protein level, suggesting that this might promote leukaemogenesis in these cases [12,14,15].

Overexpression of *HLXB9* has been recently reported in other forms of cancers, such as colorectal cancer and hepatocellular carcinoma, suggesting a common pathway of oncogenesis [16,17]. 

ChIP-on-chip studies have been undertaken to explore specific pathways of HLXB9 involvement in order to identify possible targets for this transcription factor [18]. These have shown that the latter binds to the promoter of the prostaglandin E receptor 2 gene (*PTGER2*) and decreases its expression. Comparative expression studies using microarrays have shown that t(7;12) leukaemias have a very different expression profile than MLL-positive childhood AML, to indicate that these two subsets of paediatric leukaemia are ontogenically and biologically very different [19].

**Table 1:** Total number of cases with reported rearrangements resulting in t(7;12) and/or *HLXB9-ETV6* fusion. AML, acute myeloid leukaemia; MDS, myelodysplastic syndrome; ALL, acute lymphoid leukaemia; ABL, acute biphenotypic leukaemia; AMKL, acute megakaryoblasticleukaemia; Pt no. in the second column from right refers to the patient no. as indicated in the original report.

No.	Disease	Karyotype	Pt no.	Ref.
1	T-ALL	48,XX,t(7;12)(q36;p13),+8,+19	116	[20]
2	AML-M0	47,XX,t(7;12)(q36;p13),+19	1	[14]
3	AML	48,XX,t(7;12)(q36;p13),+8,+19	2	[14]
4	AML	46,XY,t(7;12)(q36;p13)/47,idem,+8	13	[21]
5	AML-M4	47,XY,t(7;12)(q36;p13),+8	46	[22]
6	AML-M2	47,XX,t(7;12)(q36;p13),+19	1	[23]
7	AML-M5a	49,XY,t(5;7;12)(q31;q36;p13),+8,+19,+del(22)(q13)	1	[15]
8	ABL	48,XY,t(7;12)(q36;p13),+19,+22	2	[15]
9	AML-M0	48,XY,t(1;7;12)(q25;q36;p13),+8,+19	3	[15]
10	AML-M2	47,XX,t(7;12)(q36;p13),+19/49,idem,+X,+8	63	[24]
11	AML-M2	47,XX,t(7;12)(q36;p13.1),+19	64	[24]
12	AML-M6	46,XY,der(7)t(7;12)(q32;p13)del(12)(p13)/ 47,idem,+19/47,idem,+8	26	[25]
13	AML-M2	48,XX,t(7;12)(q32;p13),+13,+19	27	[25]
14	AML	47,XY,t(7;12)(q36;p13),+19	1	[7]
15	AML	48,XY,ins(12;7)(p13;q36;q11.1),+13,+19	2	[7]
16	AML	46,XY,t(7;12)(q36;p13)	1	[11]
17	AML-M1	47,XY,der(7)t(7;12)(q36;p13)del(12)(p13p13),der(12)t(7;12)(q36;p13),+19	2	[11]
18	AML-M3v	47,XY,t(7;12)(q36;p13),+19	3	[11]
19	AML	47,XX,t(7;12)(q36;p13),+19/48,idem,+19	4	[11]
20	AML	47,XX,t(7;12)(q36;p13),+19	6	[11]
21	AML	46,XX,t(7;12)(q36;p13)	9	[11]
22	AML	46,XX,t(7;12)(q32;p13)/47,idem,+19	10	[11]
23	AMKL	46,XX,add(7)(q22),del(12)(p12p13)	1	[26]
24	MDS	46,XX,der(7)t(7;12)(q22;p13)del(7)(q22q36),der(12)t(7;12)(q36;p13)	1	[8]
25	AML-M5	47,XY,del(7)(q32q35-36),t(7;12)(q36;p13),+19	2	[8]
26	AML-M1	47,XX,t(7;12)(q36;p13),+19	3	[9]
27	T-ALL	50,XX,+6,del(12)(p13),+18,+19,+22	4	[9]
28	AML-M0	47,XY,t(7;12)(q36;p13),+der(19)	5	[9]
29	AML-M4	48,XY,t(7;12)(q36;p13),+8,+19	6	[9]
30	ALL	47,XY,t(7;12)(q36;p13),+19	7	[9]
31	AML	47,XX,t(7;12)(q36;p13),+8/48,idem,+19/ 50,idem,+X,+19,+19/51,idem,+X,+8,+19,+19	17	[9]
32	AML-M0	47,XY,t(7;12)(q36;p13),+19	6	[10]
33	AML	48,XY,t(7;12)(q36;p13),+8,+19	7	[10]
34	AML-M0	46,XX,t(7;12)(q32;p13)/47,idem,+19	1	[12]
35	AML-M2	47,XX,t(7;12)(q36p13),+19	4	[12]
36	ALL	47,XX,del(7)(q31),del(12(p13)	5	[12]
37	AML-M0	47,XX,+19	6	[12]
38	AML-M5	47,XX,t(7;12)(q36p13),+19	7	[12]
39	AML	48,XY,t(7;12)(q36;p13),+8,+19	2	[19]
40	AML-M2	47,XX,t(7;12)(q36;p13),+19	3	[19]
41	AML-M0	47,XX,t(7;12)(q36;p13),+19	4	[19]
42	AML-M0	47,XX,del(7)(q11.2~21),del(12)(p13),+mar	5	[19]
43	AML-M2	47,XX,del(12)(q12),+19	6	[19]
44	AML	46,XY,inv(2)(p11p13),t(7;12)(q36;p13),der(16)t(1;16)(q22;p13),add(21)(q22)	5	[27]

It is therefore important to be able to discriminate among different types of leukaemia by setting appropriate diagnostic tests for the detection of t(7;12) rearrangements. Considering that conventional methods of chromosome banding often do not reach the resolutive power to identify translocations between chromosome ends, molecular cytogenetics represents the most accurate diagnostic method. The designing of probes with adequate specificity and sensitivity is crucial for the success of fluorescence *in situ* hybridization assays. 

In this study we describe the use of a novel three-colour FISH assay for the detection of the t(7;12). We have here demonstrated that this new approach can identify the rearrangement accurately in straight forward t(7;12) cases and also as cryptic translocation in a complex rearrangement. 

## 2. Experimental Section

### 2.1. Patients Samples

Eight patient’s samples in the form of archival methanol:acetic acid fixed chromosomes and cells suspensions were used in this study. Of these, five cases were previously reported and three new cases were contributed by the Cytogenetics laboratory of the N.N. Blokhin Russian Cancer Research Center RAMS (patients nos. 6 and 7) and the Molecular Biology Laboratory of Regional Children’s Hospital N 1 (patient no. 8). The clinical and cytogenetic characteristics of the patients are summarized in Table 2. Patients were selected on the basis of the following criteria: (i) the presence of t(7;12) (patients nos. 1–4 and 7); (ii) the presence of a fusion transcript *HLXB9-ETV6* (patient no, 5) or (iii) the presence of abnormalities of 12p and/or 7q (patients nos. 6 and 8).

**Table 2:** Clinical and cytogenetic data of the patients selected for this study.

pt	Age/sex	Disease	Karyotype	Ref.
1	7 mo/F	AML	46,XX,der(7)t(7;12)(q22;p13)del(7)(q22q36)	[8,10]
			Revised karyotype:	
			46,XX,der(7)t(7;12)(q36;p13)del(7)(q22q36)	
2	3 mo/M	AML-M0	47,XY,t(7;12)(q36;p13),+der(19)	[10]
3	5 mo/F	AML-M1	47,XX,t(7;12)(q36;p13),+19	[10]
4	8 mo/F	AML	47,XX,t(7;12),+19	[23]
5	4 mo/F	AML-M2	47,XX,t(7;16)(q36;q12),+19	[19]
			Revised karyotype:	
			47,XX,der(16)t(7;12;16)(q36;p13;q12)inv(16)(p11.2q12),+19	
6	10 mo/F	AML-M4	48,XX,+19+22,inv(16)(p13q22),del(12p)(p13)	this study
			Revised karyotype:	
			48,XX,+19+22,t(7;12)(q36;p13),inv(16)(p13q22)	
7	6 mo/F	AML-M7	47,XX,+19/idem,t(7;12)(q36;p13),+mar	this study
8	5 mo/F	MPAL	46,XX,del(7)(q11),del(12)(p13)	this study
			Revised karyotype:	
			46,XX, der(7)t(7;12)(q11;p13)del(7)(q11q36)	

Pt, patient; mo, months; y, years; M, male; F, female; AML, acute myeloid leukaemia; MPAL, mixed phenotype acute leukaemia. A revised karyotype for patients nos. 1, 5, 6 and 8 has been included after FISH analysis carried out as part of this study.

### 2.2. Probes

**Figure 1:**
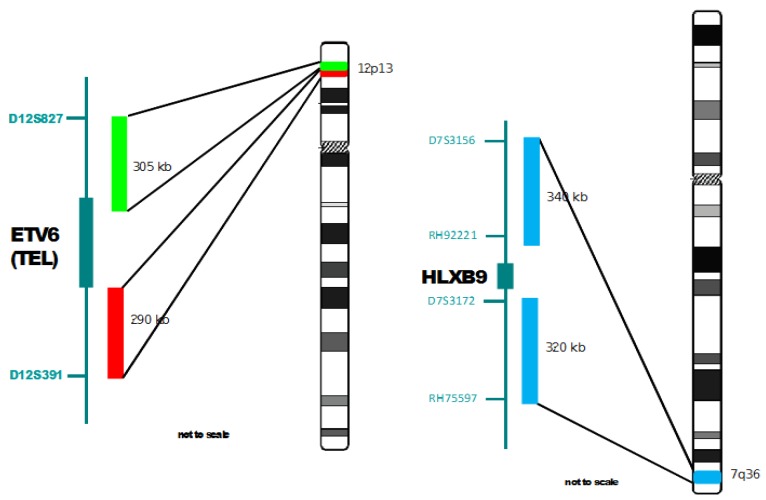
Ideograms of chromosomes 12 and 7 with details of the localization of the probes used for FISH. On the right of each chromosome ideogram, the chromosomal bands of 12p13 and 7q36 are indicated. On the left of the ideograms, the probes used and their position with respect to the gene of interest are indicated.

Probes used were: (i) a break-apart probe composed of an orange labelled probe centromeric to the *ETV6* gene in 12p13, a green labelled probe telomeric to and slightly overlapping with *ETV6* (see Figure 1); (ii) a probe combination made of two loci flanking the *HLXB9* gene, both labelled in blue (Aqua) (see Figure 1); (iii) a revised version of (i) with a more distal green probe, not to contain *ETV6* sequences (see the last Figure, Figure 8C); Probes (i) and (ii) were applied simultaneously and constituted the three colour probe set used as a novel approach in this study. This probe set, together with (iii) were designed and provided by MetaSystems GmbH, Altlussheim, Germany. Additional probes used were: (iv) a whole chromosome painting probe specific for chomosome 12 (12WCP), labelled with biotin and detected with Cy3-streptavidin and (v) a whole chromosome painting specific for chromosome 16 (16WCP), labelled with FITC. Probes (iv) and (v) were purchased from Cambio, Cambridge, UK.

### 2.3. Fluorescence *in Situ* Hybridization

Fluorescence *in situ* hybridization (FISH) experiments were carried out according to the manufacturers instructions with slight modifications. Briefly: slides were denatured with 70% formamide at 70 °C for 5 min. Probe mixture was denatured at 65 °C for 10 min, incubated at 37 °C for 10 min, and subsequently applied to the slides under a 22 × 22 mm cover-slip. After overnight hybridization, slides were washed with 2× SSC (pH 8.0) for 5 min on shaker, followed by another wash in 4× SSC/Tween20 for 5 min on shaker and then a final wash in 1× PBS for 5 min on shaker. The slides were then mounted in Vectashield (Vector Laboratories Ltd., Peterborough, UK) containing 49, 6-Diamidine-29-phenylindole dihydrochloride (DAPI). Hybridized chromosomes and nuclei were viewed and images were captured using a Zeiss axioplan epifluorescence microscope (Carl Zeiss, Cambridge, UK) equipped with a CCD camera and MetaSystems Isis v. 5.3 software.

## 3. Results

**Figure 2:**
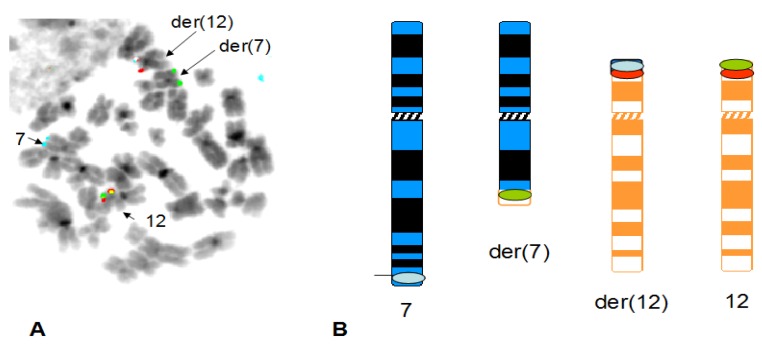
Detection of t(7;12) rearrangement with simultaneous deletion of chromosome 7 at band q22. A representative FISH image obtained on a bone marrow metaphase of patient 1 shows localization of FISH signals on chromosome 7 (blue signals), der(7) (green signals), chromosome 12 (green and orange signals) and der(12) (blue and orange signals. Note that the the der(7) is considerably shorter than the normal chromosome 7, indicating a deletion of the long arm. The DAPI counterstain used to visualize the chromosomes has been converted into grayscale to simulate a G-like banding pattern (**A**). The schematic representation of the hybridization pattern is also shown on the ideograms, that depict the deletion on the der(7) at band q22 (**B**).

**Figure 3:**
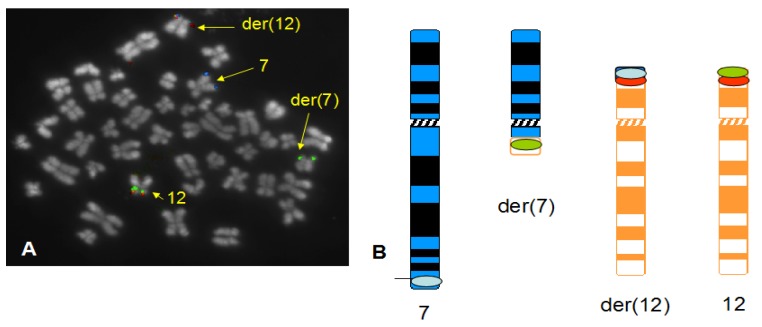
Detection of t(7;12) rearrangement with simultaneous deletion of chromosome 7 at band q11. A representative FISH image obtained on a bone marrow metaphase of patient 8 shows localization of FISH signals on chromosome 7 (blue signals), der(7) (green signals), chromosome 12 (green and orange signals) and der(12) (blue and orange signals). Note that the the der(7) is considerably shorter than the normal chromosome 7, indicating a deletion of the long arm. The DAPI counterstain used to visualize the chromosomes has been converted into grayscale to simulate a Q-like banding pattern (**A**). The schematic representation of the hybridization pattern is also shown on the ideograms, that depict the deletion on the der(7) at band q11 (**B**).

A total of eight patients were analysed in this study. Of these, five patients had previously been reported. Patients nos. 1–4 had already been studied for the presence of the t(7;12) [8,10,23], whereas patient no. 5 failed to show a t(7;12) in previous reports [19], although this patient sample showed an ectopic expression of *HLXB9* and a fusion transcript *HLXB9-ETV6*. Patients nos. 6–8 were not reported previously. Morphological and cytogenetic characteristics of all patients are summarized in Table 2. FISH studies were carried out using a new three-colour probe set on all patients. Furthermore, patient no. 5 was investigated using whole chromosome 16 paint (wcp16) and whole chromosome 12 paint (wcp12) simultaneously. In addition to these probes, a revised version of the *ETV6* dual colour probe was also applied at a later stage to get a better definition of the breakpoint on 12p13. The new three colour probe set identified the t(7;12)(q36;p13) in seven patients out of eight. Of these, four patients (patients nos. 1–4) were already investigated in previous studies and were used as positive controls in this work. Interestingly, patients nos. 1 and 8 both showed a deletion of 7q in the same chromosome involved in the t(7;12) rearrangement (see Figure 2 and Figure 3), although the deletion breakpoint in patient no. 8 seems more proximal. The t(7;12) rearrangement in patient no. 8 should be then revised as der(7)t(7;12)(q36;p13)del(7)(q11q36). Similarly, the rearrangement in patient no. 1 could be further refined as der(7)t(7;12)(q36;p13)del(7)(q22q36). 

**Figure 4:**
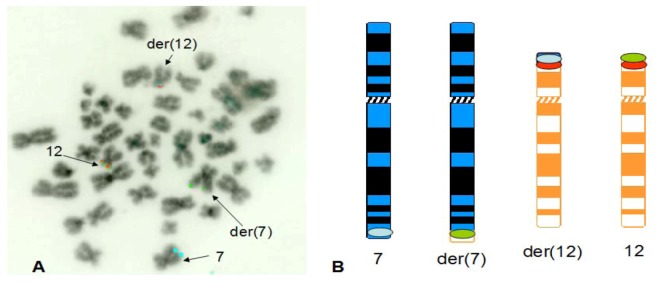
Detection of straight forward t(7;12) rearrangement. A representative FISH image obtained on a bone marrow metaphase of patient 2 shows localization of FISH signals on chromosome 7 (blue signals), der(7) (green signals), chromosome 12 (green and orange signals) and der(12) (blue and orange signals). The DAPI counterstain used to visualize the chromosomes has been converted into grayscale to simulate a G-like banding pattern (**A**). The schematic representation of the hybridization pattern is also shown on the ideograms (**B**).

The t(7;12) rearrangement in patients nos. 2–4 was successfully confirmed by FISH using the three colour probe set (a representative FISH image from patient 2 only is shown in Figure 4A). In these cases, same as for patients nos. 1 and 8, it is possible to appreciate a clear split of the *ETV6* probe, with a green signal localized on the der(7) and an orange signal localized on the der(12), proximal to the blue signal indicating translocation of the *HLXB9* region from chromosome 7.

**Figure 5:**
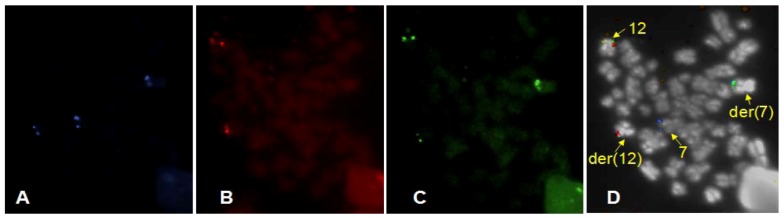
Detection of t(7;12) rearrangement shows heterogeneity of breakpoints in the *ETV6* and *HLXB9* regions. A representative FISH image obtained on a bone marrow metaphase of patient 7 shows localization of blue hybridization signals on chromosome 7, the der(7) and the der(12) (**A**), orange hybridization signals on chromosome 12 and the der(12) (**B**) and green hybridization signals on chromosome 12, the der(12) and the der(7) (**C**). Images relative to all fluorophores have been merged with the DAPI stained metaphase image to show co-localization of FISH signals. The DAPI counterstain has been converted into grayscale to simulate a Q-like banding pattern (**D**).

A different situation has been observed for patients nos. 6 and 7, where the three colour probe set has shown a different distribution of hybridization signals, with the orange signals, specific for the probe proximal to *ETV6*, present on both normal chromosome 12 and der(12), the green signals, specific for the probe distal to (and overlapping with) *ETV6*, present in three locations [normal 12, der(12) and der(7)], and blue signals also present in three locations [normal 7, der(7) and der(12)]. This hybridization pattern results in the two derivatives carrying signals for all three fluorophores (see Figure 5). 

**Figure 6:**
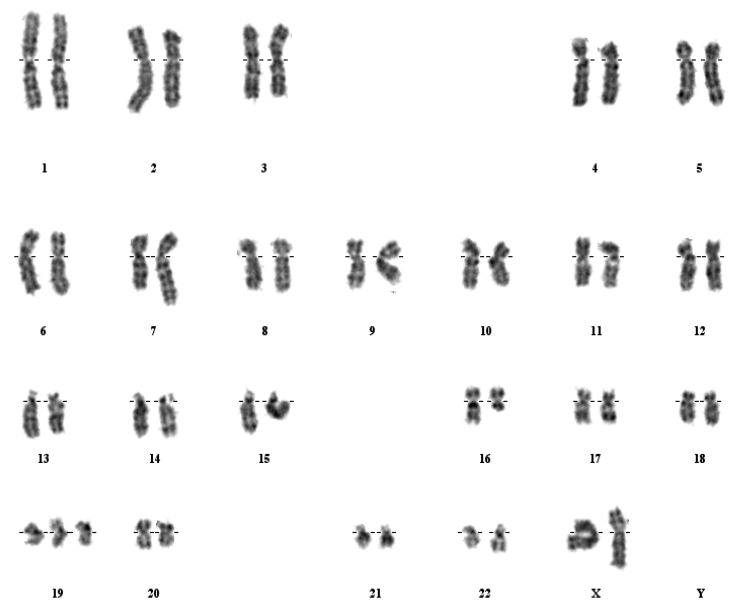
The t(7;16) rearrangement in patient no. 5. A representative karyotype obtained after G-banding of bone marrow metaphase of patient no. 5 shows a t(7;16), with a shorter chromosome 16, der(16), and an elongated chromosome 7, der(7). No involvement of chromosome 12 is noted at this stage.

The remaining patient (no. 5) was selected on the basis of its expression profile, indicative for the presence of the t(7;12), although this rearrangement was not seen at the cytogenetic level [19]. Patient no. 5 was reported as having a translocation t(7;16), as shown by G-banding analysis (see Figure 6).

**Figure 7:**
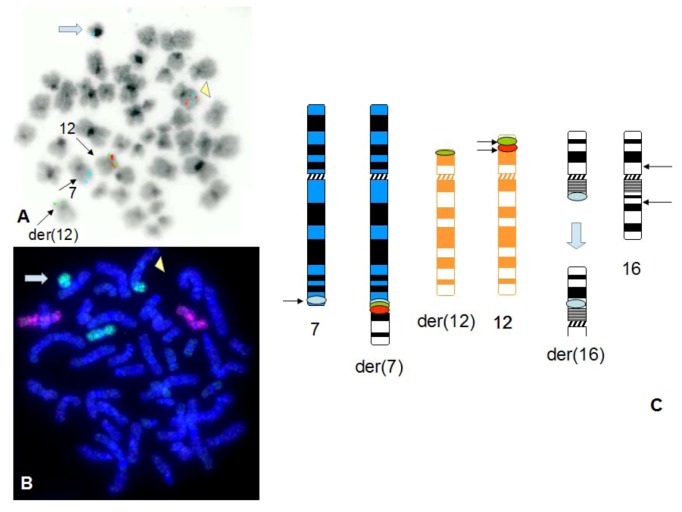
Complex cryptic three way rearrangement t(7;12;16). Three colour FISH using the *ETV6-HLXB9* probe set on a bone marrow metaphase of patient no. 5 shows localization of blue hybridization signals on chromosome 7, the der(7), the der(12) and the der(16), whereas orange and green hybridization signals are localized on chromosome 12 and the der(7) (**A**). The use of whole chromosome paint did not confirm the involvement of chromosome 12, due to the small size of the translocated region. However, the whole chromosome paint specific for chromosome 16 highlighted the involvement of this chromosome and its translocation onto chromosome 7 (**B**). The der(7) is indicated by a yellow arrowhead and the der(16) is indicated by a block arrow in light blue in both (**A**) and (**B**). The schematic representation in (**C**) shows localization of FISH signals on the ideograms of the respective chromosomes involved in the rearrangement. This also shows a possible inversion event to justify localization of blue hybridization signals on what appears to be the short arm of chromosome 16.

**Figure 8:**
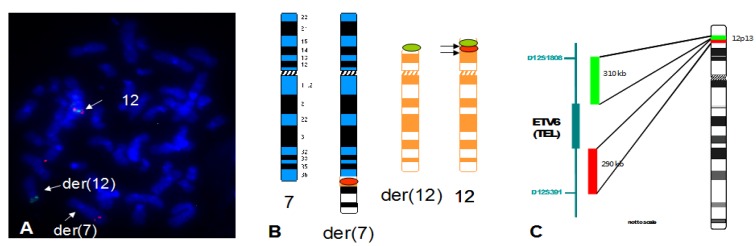
Refined breakpoint localization using a re-designed probe set for the *ETV6* region. A representative FISH image obtained on a bone marrow metaphase of patient 5 shows refined localization of breakpoints, with der(7) carrying only orange signals, der(12) carrying only green signals and chromosome 12 carrying both green and orange signals (**A**). This is also shown in the schematic representation depicting ideograms of the chromosomes hybridized (**B**). The newly observed hybridization pattern is due to the green probe being more distal than in the previous probe set, hence non-overlapping with *ETV6* sequences (**C**).

However, this appeared to be a more complex rearrangement involving a cryptic t(7;12) in the form of t(7;12;16) (see Figure 7). The use of WCP16 confirmed that the terminal portion of the der(7) was chromosome 16 material. Chromosome 12 insertion into the der(7) was not shown by WCP12. However, the three-colour probe set showed that signals corresponding to the three fluorophores were present on the der(7) (Figure 7). Only orange signals were present on the der(7) when using the new version of the break-apart *ETV6* probe (see Figure 8). In the revised version, the green labelled probe was designed to be distal to the one in the previous version and would not overlap with *ETV6* sequences. The blue hybridization signals corresponding to the *HLXB9* region were found on the normal chromosome 7 and the der(16) (see Figure 7).

## 4. Discussion

### 4.1. Summary of Results on Chromosome 7 and 12 Breakpoints

The use of our novel three colour FISH probe set has enabled the detection of a t(7;12) rearrangement in all 8 patients here reported. In five cases (patients nos. 1–4 and 8), the breakpoints were within the *ETV6* gene in 12p13 between the orange probe and the green probe and proximal to the *HLXB9* region in 7q36, targeted by the blue probe. In patients nos. 5, 6 and 7 the breakpoint at 12p13 occurred also within *ETV6*, but in a region distal to the one reported in the other patients. This resulted in a different pattern of FISH signals, with blue, orange and green signals on the der(12) (patients nos 6 and 7) or on the der(7) (patient no. 5). The new version of the break-apart probe is designed to cover a broader area, to include a range of breakpoints in 12p13 within the *ETV6* region. Heterogeneity in the breakpoint regions of both 12p13 and 7q36 has been previously reported [7,10,28]. This is obviously demonstrated once again in this report, where the variability not only in 12p13, but also in the 7q36 breakpoints is reiterated through the observation of the different patterns in different patients. In particular, whereas most patients show two blue signals (one on the normal chromosome 7 and one on the derivative), patients nos. 5 and 7 show blue hybridization signals in three locations (see Figure 3 and Figure 4). The use of such a versatile probe-set would enable the detection of t(7;12) in a larger number of patients, to include those with breakpoints in the area of interest, but not necessarily within the genes of interest. Altogether, the new approach would make interpretation of FISH images easier and more immediate in the diagnostic setting.

### 4.2. Finding of a New Cryptic t(7;12) Rearrangement

This is the third report to date of a cryptic t(7;12) rearrangement as part of a complex translocation involving more than two chromosomes. The patient here described had been reported as having a t(7;16). As part of a previous screening for the t(7;12) on infant leukaemias with chromosome 7 abnormalities, FISH analysis was performed by us using a different set of probes for *ETV6*. However, this failed to show a rearrangement of chromosome 12 (data not shown). Later on, this patient was reported to have an *HLXB9-ETV6* fusion transcript at the molecular level [19]. This prompted us to repeat the FISH analysis using the novel three-colour probe set here described. The latter approach revealed a t(7;12) rearrangement that was confirmed as a three way translocation. From our observations, it became obvious that the probes designed to flank the regions of interests in 7q36 and 12p13 respectively, allowed the detection of a broader spectrum of breakpoints within these areas. To the best of our knowledge, only two cases of a cryptic t(7;12) translocation have been reported in the literature [15]. None of these cases involved chromosome 16 as they had a t(5;7;12) and a t(1;7;12) respectively. Both these cases had over-expression of *HLXB9*, suggesting that the mechanism of leukaemogenesis in the t(7;12) cases and variants might be similar. 

The presence of variant or masked translocations has been reported in numerous instances in leukaemia. Classic examples of these rearrangements are variant or masked Philadelphia chromosomes in chronic myeloid leukaemia [29,30], variant or complex t(15;17) in acute promyelocytic leukaemia [31,32] and many others. Complex translocations involving *ETV6* resulting in known fusions have also been reported [15,33,34,35]. The mechanisms that lead to the formation of these complex rearrangements are still unclear, as still unclear is the sequence of events that occurs in double strand breaks involving more than two chromosomes. In the complex translocation presented in this report, the hybridisation pattern observed is suggestive of insertion of chromosome 12 material into the derivative 7, translocation of chromosome 16 material onto the derivative 7 and also possibly an inversion or an insertion in the der(16) to justify the presence of blue signals in what appears to be its short arm. This is indicative of at least two breakpoints on chromosome 16 (see Figure 7). Further analysis with informative FISH probes might help to fully elucidate this particular case. Nevertheless, the importance of disclosing cryptic translocations and complex rearrangements remains in the potential prognostic value attributable to it. Although the prognostic significance of the t(7;12) merits further investigation, it is to date believed that this rearrangement is an indicator of poor clinical outcome [11]. The t(7;12) is also the second most common chromosomal abnormality in infant leukaemia after mixed-lineage leukaemia gene (*MLL*) rearrangements [6]. It is therefore relevant to be able to discriminate between two very different parameters on which to base therapy choices. The availability of a good FISH probe-set to be used for screening in the diagnostic setting is at the basis of patient stratification into low-risk/high risk categories. The finding of cryptic t(7;12) indicates that the incidence of these rearrangements might be higher than reported to date. The screening of leukaemia patients using the new FISH assay will help define the real incidence of these cases.

## 5. Conclusions

Our study showed the effectiveness of a novel three colour FISH approach for the detection of t(7;12) rearrangements involving the *HLXB9* region in 7q36 and the *ETV6* region in 12p13. Abnormalities of these regions are often undetected by conventional cytogenetic methods. We foresee the usefulness of such FISH assay in the diagnostic setting, for a more effective screening of specific rearrangements whose incidence might be to date underestimated. 
